# Classification of the cystic duct patterns and endoscopic transpapillary cannulation of the gallbladder to prevent post-ERCP cholecystitis

**DOI:** 10.1186/s12876-019-1053-6

**Published:** 2019-08-05

**Authors:** Jun Cao, Xiwei Ding, Han Wu, Yonghua Shen, Ruhua Zheng, Chunyan Peng, Lei Wang, Xiaoping Zou

**Affiliations:** Department of Gastroenterology, Nanjing Drum Tower Hospital, The Affiliated Hospital of Nanjing University Medical School, Zhongshan Road 321, Nanjing, 210008 Jiang Su Province China

**Keywords:** Cystic duct, Classification, Cholecystitis, Endoscopic transpapillary gallbladder cannulation, Endoscopic retrograde cholangiopancreatogram

## Abstract

**Background:**

Endoscopic transpapillary cannulation of the gallbladder is useful but challenging. This study aimed to investigate cystic duct anatomy patterns, which may guide cystic duct cannulation.

**Methods:**

A total of 226 patients who underwent endoscopic transpapillary cannulation of the gallbladder were analyzed retrospectively.

**Results:**

According to the cystic duct take-off, 226 cystic duct patterns were divided into 3 patterns: Type I (193, 85.4%), located on the right and angled up; Type II (7, 3.1%), located on the right and angled down; and Type III (26, 11.5%), located on the left and angled up. Type I was further divided into three subtypes: Line type, S type (S1, not surrounding the common bile duct; S2, surrounding the common bile duct), and α type (α1, forward α; α2, reverse α). Types I and III cystic ducts were easier to be cannulated with a higher success rate (85.1 and 86.4%, respectively) compared with Type II cystic duct (75%) despite no statistically significant difference. The reasons for the failure of gallbladder cannulation included invisible cyst duct take-off, severe cyst duct stenosis, impacted stones in cyst duct or neck of the gallbladder, sharply angled cyst duct, and markedly dilated cyst duct with the tortuous valves of Heister.

**Conclusion:**

Classification of cystic duct patterns was helpful in guiding endoscopic transpapillary gallbladder cannulation.

## Background

Endoscopic retrograde cholangiopancreatography (ERCP) is a minimally invasive modality widely used for diagnosing and treating pancreaticobiliary diseases. However, it can cause several serious adverse events, such as post-ERCP pancreatitis (PEP), perforation, bleeding and post-ERCP cholecystitis (PEC). Although a large number of studies focused on PEP, a few were performed on PEC. Freemen et al. showed that the incidence of cholecystitis was 0.5% 16 days after ERCP [1]. Lee et al. reported that the incidence of cholecystitis was 17% after endoscopic common bile duct stone removal during an average 18-month follow-up [2]. A previous study demonstrated that the incidence of PEC was 1.35% (36/2672) within 2 weeks after ERCP [3]. The study further identified high-risk factors for PEC like history of acute pancreatitis and chronic cholecystitis, gallbladder opacification, et al. Though rare, PEC may cause serious consequences especially in patients with multiple comorbidities. Therefore, it needs to be prevented in high-risk patients during ERCP.

Endoscopic gallbladder drainage has been used for patients with acute cholecystitis unfit for surgery due to multiple comorbidities [4–7]. In 1984, Kozarek first reported endoscopic transpapillary cannulation of the gallbladder [8]. Endoscopic transpapillary gallbladder drainage (ETGD) is a safe and effective method for decompressing the gallbladder and alleviating the inflammation [7, 9]. Nasogallbladder drainage can directly observe the biliary drainage output and rinse the gallbladder [5]. However, ETGD is a technically challenging method. Cholecystitis becomes more severe if gallbladder drainage is not performed due to the failure of cystic duct cannulation following the injection of contrast agents into the gallbladder. It is presumed that cystic duct patterns may influence on the success rate of transpapillary cannulation of the gallbladder. To date, a few studies investigated the cystic duct patterns. Further, no published study analyzed the impact of cystic duct patterns on the success rate of gallbladder cannulation.

In this study, cystic ducts from 226 patients were observed, analyzed and classified into different patterns according to their anatomy. The study also examined the impact of this classification on the success rate of gallbladder cannulation.

## Methods

### Ethical statement

This study conformed to the ethical guidelines of the 2013 Declaration of Helsinki and Strengthening the Reporting of Observational Studies in Epidemiology guidelines. The study was approved by the Ethical Committee at Nanjing Drum Tower Hospital (study number 2017–167-01).

### Patients

The medical records of patients with gallbladder in situ and high-risk factors for PEC undergoing ERCP in our hospital from December 2016 to February 2018, were reviewed and analyzed retrospectively. Two senior endoscopists independently observed cystic duct patterns directly under a cholangiogram. If they had different opinions, the third experienced endoscopist decided the pattern of the cystic duct.

### Endoscopy protocol

Duodenal side-viewing endoscopes (JF-260, TJF-240, or TJF-260, Olympus, Tokyo, Japan) were used to perform the ERCP. The patients with pancreatibiliary diseases underwent ERCP using conventional devices. After successful bile duct cannulation, a hydrophilic guidewire (260 cm long, 0.035-in. wide) with a flexible end was used to pass through the valves of Heister or tortuous cystic ducts and cannulate into the cystic duct. Once the guidewire had made a generous loop in the gallbladder, the guiding catheter (sphincterotome, extraction balloon, or other catheter) was advanced, and bile was aspirated to confirm the position [10]. Subsequently, a longer guidewire (0.035-in. wide) was exchanged into the gallbladder and a 5- to 7-Fr nasobiliary tube (Olympus Medical System) or plastic stents with different diameters were inserted and left indwelling in the gallbladder over the guidewire.

### Statistical analysis

The deviation of the data with the normal distribution of variables was described as mean ± standard deviation. Frequency was used to describe the classification of variables. The differences between different types or subtypes were compared using the Fisher exact test. A *P* value less than 0.05 was considered statistically significant.

## Results

### Patient characteristics

A total of 226 patients were examined. The number of patients with common bile duct stones was 218 (215 with chronic cholecystitis and cholecystolithiasis and 3 with Mirizzi syndrome). Further, one had hilar cholangiocarcinoma, three had common bile duct strictures resulting from pancreatic head cancer, one had distal cholangiocarcinoma, and three had major duodenal papillary carcinomas. These patients with gallbladder in situ underwent ERCP for the pancreaticobiliary disease. The mean age of the patients was 59.50 ± 16.16 years (range, 8–92 years). Also, 88 of 226 patients (38.9%) were female.

### Different types of cystic duct patterns

**Fig. 1:**
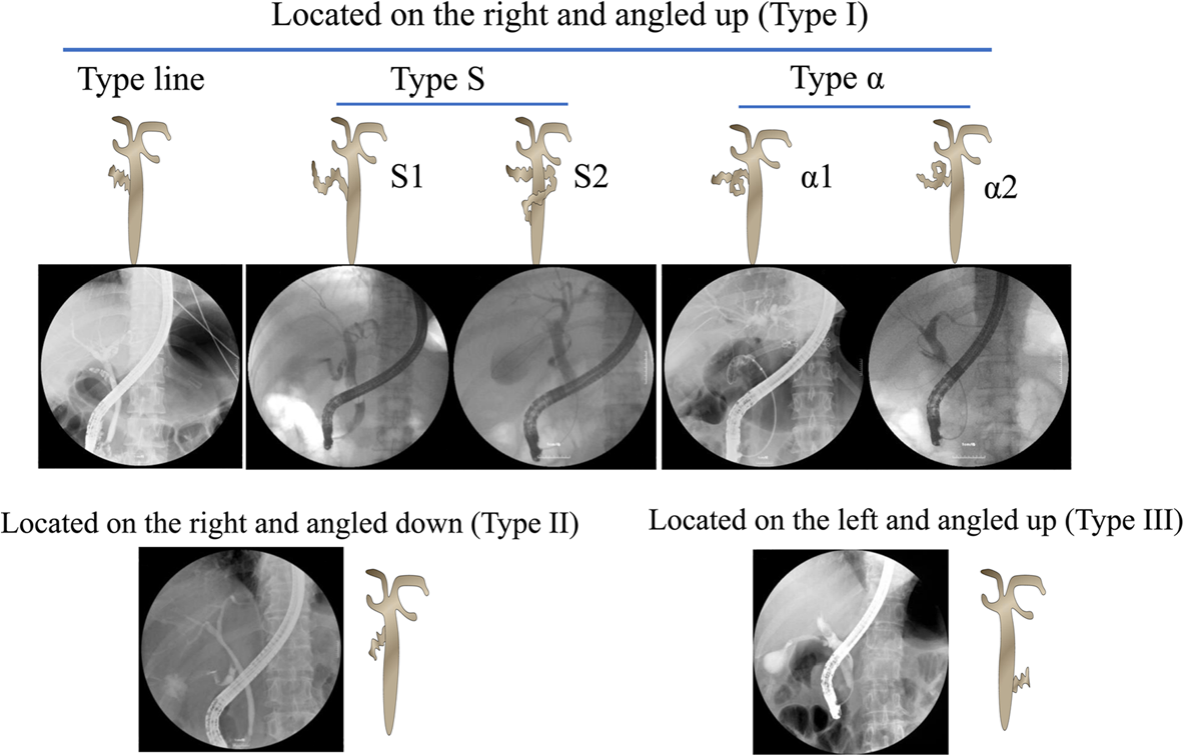
Schematic diagram of different types of cystic duct patterns

Itoi et al. have divided cystic ducts into three patterns [11]: Type I, angled up and located on the right; Type II, angled down and located on the right; and Type III, angled up and located on the left (low confluence of the cystic duct). Type I pattern was found to have great variation and could be further divided into three subtypes: linear type, S type (S1, not surrounding common bile duct; S2, surrounding common bile duct), and α type (α1, forward α; α2, reverse α) (Fig. 1). In the present study, all cystic duct patterns were Type I (193, 85.4%), Type II (7, 3.1%), and Type III (26, 11.5%). The linear type was found in 85 patients and accounted for 52.8% (85/161) of the Type I pattern (Table 1).

**Table 1:** Different subtypes of Type I cystic duct pattern

	Type I pattern				
Configuration	Line	α		S	
		α1	α2	S1	S2
Number of patients (N)	104	18	17	48	6

### Success rate of gallbladder cannulation

The gallbladder cannulation was performed in patients with a high risk of PEC according to the cystic duct patterns. The patient’ characteristics with different factors determining the high risk of PEC are described in Table 2. The overall success rate of cystic duct cannulation was 85.1% (171/201). Types I and III cystic ducts were easier to cannulate with a high success rate (85.1 and 86.4%, respectively). The success rate of cannulation in Type II was the lowest (75%). However, no statistically significant difference was found among different types or subtypes of the cystic duct (Tables 3 and 4).

**Table 2:** Patients’ characteristics with different PEC Risk factors

	I					II	III
	Line	S1	S2	α1	α2		
Chronic cholecystitis (*N*)	83	32	2	16	17	3	17
Gallbladder opacification (*N*)	24	17	1	5	4	0	11
High leukocyte count before ERCP (*N*)	13	3	2	2	3	3	2
History of acute pancreatitis (*N*)	3	1	0	1	0	0	1

**Table 3:** Success rate of cannulation for different subtypes of type I cyst ducts

	Subtypes of I pattern					*P*
	Line	α		S		
		α1	α2	S1	S2	
Number of patients (*N*)	96	18	17	39	5	
Number of successful cannulation (*N*)	87	16	14	28	4	0.066
Success rate of cannulation (%)	90.6%	88.9%	82.4%	71.8%	80.0%

**Table 4:** Success rate of cannulation for three types of cyst ducts

	Cyst ducts pattern			*P*
	Type I	Type II	Type III	
Number of patients (N)	175	4	22	
Number of successful cannulation (*N*)	149	3	19	
Success rate of cannulation (%)	85.1%	75.0%	86.4%	0.84

### Complications

Three patients had abdominal pain after ERCP (two in the α1 group and one in the S1 group), and one had mild hematobilia in the α2 group. All cases with complication recovered after conservative treatment. Because the low incidence of complication rates in different groups, we did not make a comparison between the different groups. One right epigastric pain was suspected to be related to cystic duct cannulation.

## Discussion

The endoscopic gallbladder drainage technique is challenging for endoscopists after successful bile duct cannulation. Previous studies reported the technical success rate of gallbladder cannulation as 70.6–90%, but these studies had small sample size (18–34 patients) [4, 12, 13]. Successful endoscopic transpapillary cannulation of the gallbladder depends on a complete understanding of the anatomy and the possibility of stenosis or obstruction of the cystic duct. Usually, cystic ducts were divided into three patterns as we described above. We found that Type I pattern has great variation and could be further divided into three subtypes: linear type, S type (S1, not surrounding the common bile duct; S2, surrounding the common bile duct), and α type (α1, forward α; α2, reverse α) (Fig. 1). The Type I cystic duct was the most common and accounted for 85.4% in 226 cystic duct patterns. The linear type cystic duct accounted for more than half in the three subtypes of the Type I pattern (Table 2).

**Fig. 2:**
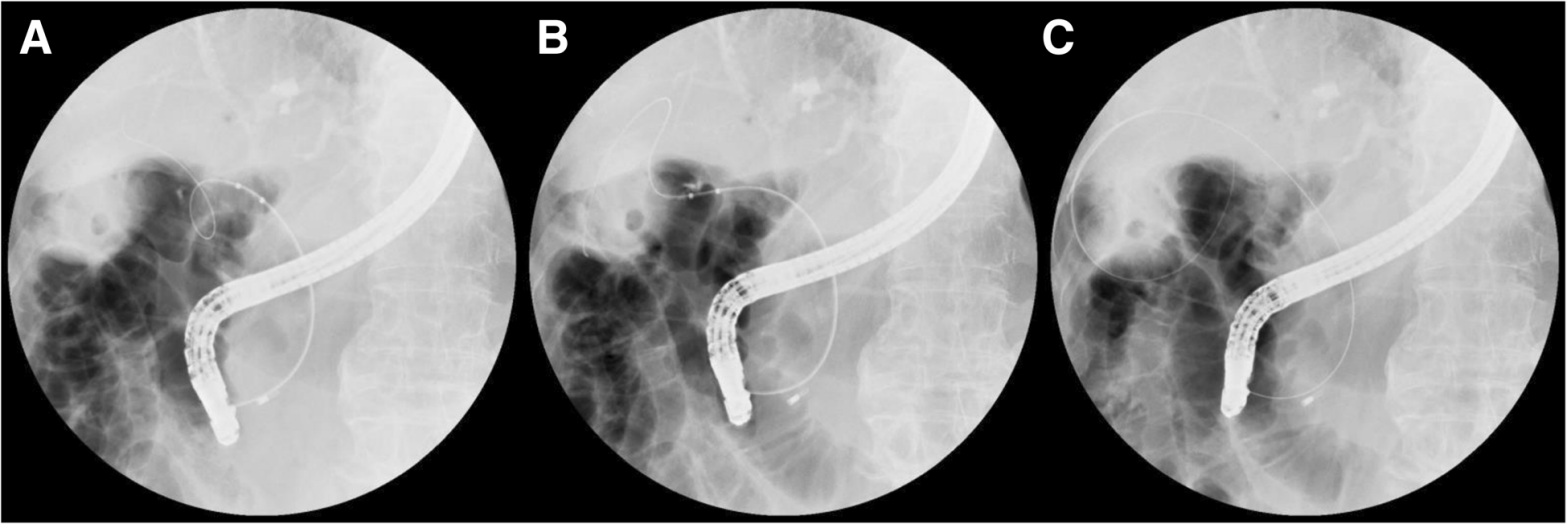
Subtype α2 of cystic duct became the subtype line one using the inflated dilation catheter. (**a**) The guidewire passed through the subtype α cystic duct into the gallbladder. (**b**) The guidewire was straightened using the inflated dilation catheter. (**c**) The looped guidewire passed into the gallbladder

Gallbladder cannulation requires specific skills due to the origin of cystic duct take-off, cystic duct configuration, corkscrew profile of the cystic duct, and presence of valves of Heister. Performing gallbladder cannulation according to the classification of cystic duct patterns is relatively easy and safe. Therefore, the first step in endoscopic transpapillary cannulation of the gallbladder is to accurately define the origin of cystic duct take-off [7]. During ERCP, the bile duct is selectively cannulated to obtain a direct cholangiogram. The cystic duct can be opacified during a cholangiogram, but if it fails to be opacified owing to cystic duct obstruction, some contrast can be injected under pressure to allow filling of the cystic duct using an occlusion balloon cholangiogram [4]. Also, a small amount of contrast medium is injected into the common bile duct to avoid filling the gallbladder with a further increase in intraluminal pressure [10]. Then, a 0.035-in., 260-cm-long hydrophilic guidewire with a flexible end is useful for seeking the cystic duct take-off. Based on different patterns of the cystic duct, different accessories are selected to cannulate the duct into the gallbladder. The use of a sphincterotome or a catheter with a flexible tip generally helps in cystic duct cannulation because it bows toward the cystic duct in a right-sided take-off (Types I and II). Since Type III is located on the left and angled up, a rotatable sphincterotome is particularly helpful if a standard sphincterotome fails. This study found that for subtypes S1 and α of Type I, tortuous cystic ducts were straightened and became linear cystic ducts by pulling the occlusion balloon backward in the cystic duct take-off (Fig. 2). This prevented the cystic duct from mechanical injury. If tortuous cystic ducts cannot be straightened, it is better to choose a 5-Fr soft catheter so as to prevent the cystic duct from mechanical injury and deeply cannulate into the gallbladder. The close coordination between the catheter and the guidewire should also be given special attention during endoscopic transpapillary cannulation of the gallbladder.

The success rate of cystic duct cannulation in previous reports was up to 90% (26/29), but these studies had small sample size. In the present study, cystic duct cannulation was performed in 201 patients with an overall success rate of 85.1%. No statistically significant difference was observed among different types or subtypes of the cystic ducts. The results suggested that more patients should be examined to elucidate further which type of cystic duct is easier to cannulate.

**Fig. 3:**
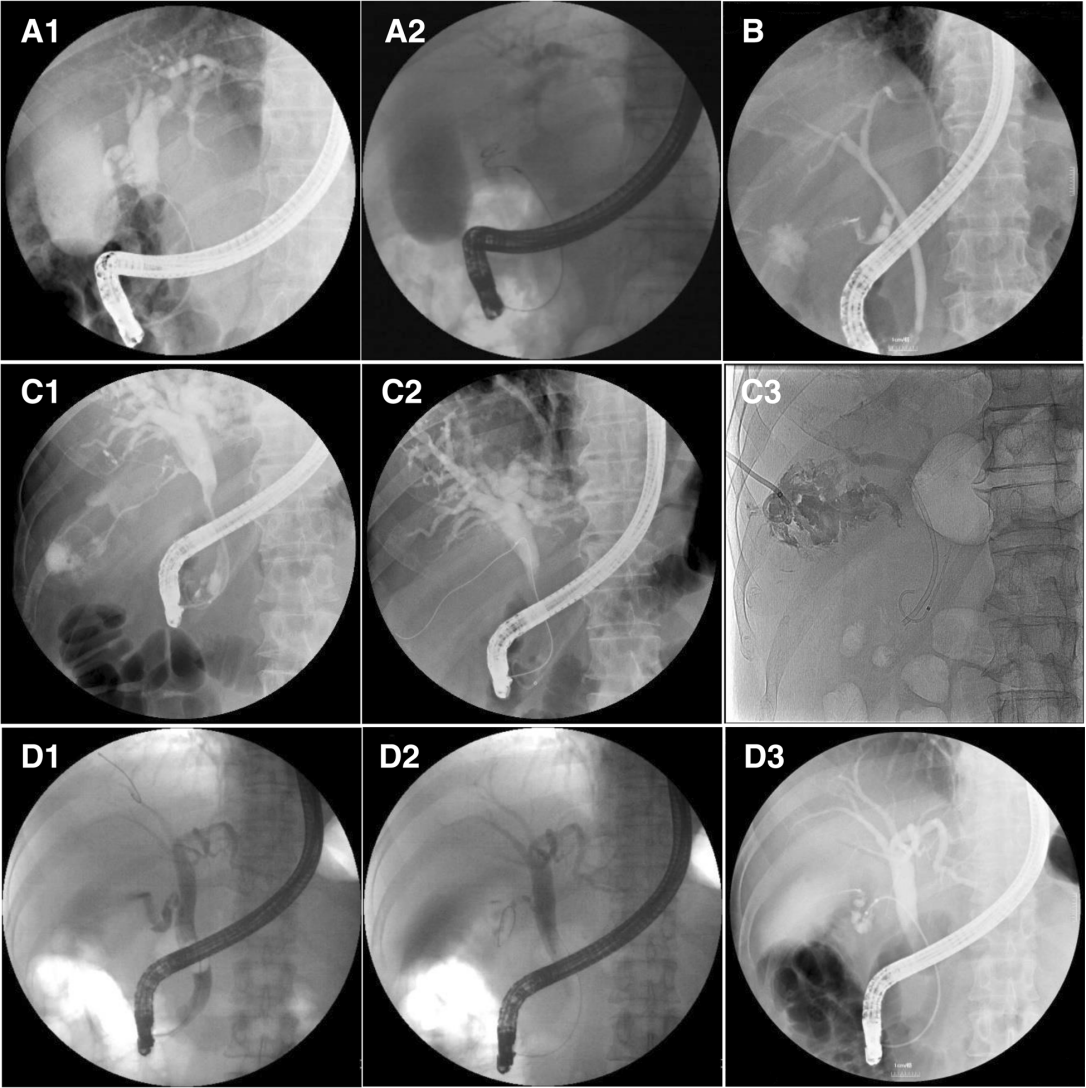
Representative cases of failure of endoscopic transpapillary gallbladder cannulation. (**a**) The dilated duct looked like a corkscrew duct owing to swollen valves of Heister; a spiraled guidewire in the dilated duct with swollen valves of Heister. (**b**) Type II cystic duct impacted the stone in the cystic duct take-off. (**c**) The duct with stenosis; the guidewire passed through the cystic duct but the 5-Fr catheter failed. (**d**) Subtype S1 cystic duct; the guidewire and the 5-Fr catheter failed to pass through the angled cystic duct

Several factors accounted for the technical failure of endoscopic transpapillary gallbladder cannulation (Fig. 3): invisible cystic duct take-off on cholangiography resulting from impacted stones in the cystic duct or the neck of the gallbladder, severe cystic duct stenosis resulting from chronic inflammation or neoplastic invasion, sharply angled cystic duct, and markedly dilated cystic duct with the tortuous valves of Heister like a corkscrew [5]. These factors might block the advancement of the guidewire or make it difficult to traverse with the guidewire. Occasionally, although the guidewire passed through the narrow cystic duct, the catheter could not pass through it owing to its narrow caliber.

**Fig. 4:**
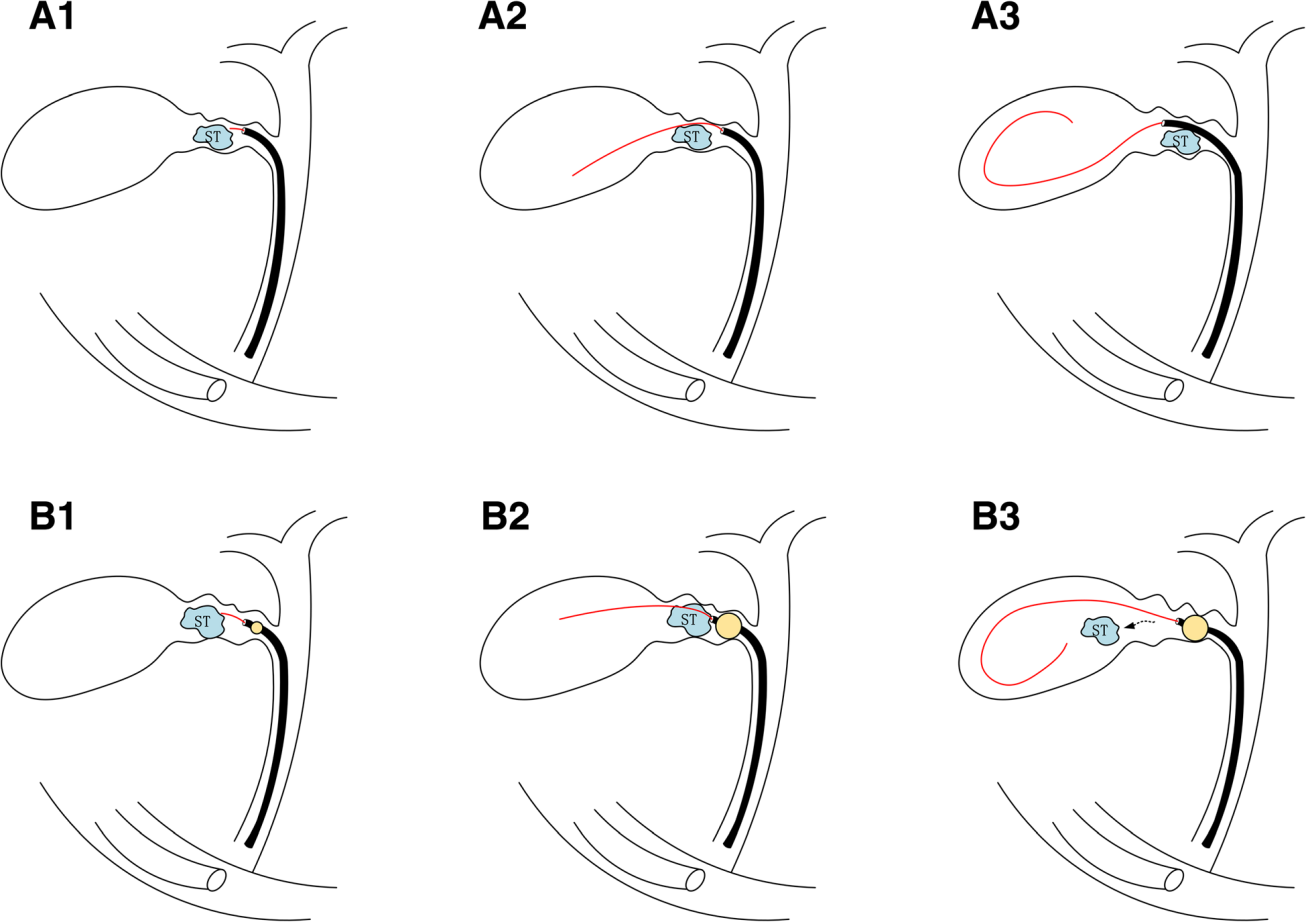
Cystic duct cannulation when a stone impacted in the neck of the gallbladder. (A1) Access to the cystic duct using the ERCP catheter with a guidewire. (A2–3) The impacted stone (ST) was bypassed using the guidewire/catheter. (B1) Access to the cystic duct using the dilation catheter with a guidewire. (B2–3) The impacted stones (ST) were dislodged into the gallbladder using the dilation inflated cathete

If impacted stones in the cystic duct or the neck of the gallbladder were confirmed under fluoroscopic control, it was better to choose a dilation balloon catheter for cystic duct cannulation. If a guidewire was passed beyond the obstruction with several attempts and inserted into the gallbladder, the catheter was sometimes bypassed, impacting the stone into the gallbladder [9] (Fig. 4a). If the catheter or guidewire could not be inserted into the gallbladder, the cystic duct was cannulated into a gallbladder by pushing forward or pulling backward the inflated dilation balloon (Fig. 4b). The impacted stone was either dislodged into the gallbladder or simply bypassed by pushing forward or pulling backward the inflated dilation balloon [4]. If cystic duct stenosis is severe, a hydrophilic guidewire through the valves of Heister is sometimes advanced into the cystic duct, but it is difficult to introduce even a 5-Fr catheter into the gallbladder through the cystic duct with stenosis. Performing cannulation in the cystic duct with a sharp angle using the balloon occlusion technique is usually not difficult. Sometimes, deep cannulation is very difficult because a sharply angled cystic duct cannot be straightened and the guidewire cannot be passed easily using the balloon occlusion technique. Moreover, the markedly dilated cystic duct resulting from the take-off obstruction due to an impacted stone or stenosis looks like a corkscrew duct owing to the tortuous, edematous valves of Heister. Cannulation cannot be performed because the guidewire also becomes a spiral one in the dilated cystic duct with swollen valves of Heister.

The cystic duct perforation or hemorrhage may occur with guidewire or catheter manipulation. However, no perforation or severe hemorrhage related to cystic duct cannulation occurred in the present study. A major complication was right epigastric pain. Therefore, cystic duct cannulation was a relatively safe procedure.

## Conclusions

With the increase in endoscopic gallbladder drainage for elderly patients with multiple comorbidities at a high risk of cholecystectomy, it has become essential to understand the classification of cystic duct patterns. Successfully and safely performing endoscopic transpapillary cannulation of the gallbladder according to the classification of cystic duct patterns is extremely helpful. Endoscopic gallbladder drainage may be a prophylactic measure during ERCP to prevent the occurrence of PEC.

## Data Availability

The data set analyzed in the current study cannot be opened to public because patients’ privacy must be protected and IRB does not permit to do so. However, data are available from the author upon reasonable request.

## Abbreviations

ENBD: nasobiliary drainage
ERCP: endoscopic retrograde cholangiopancreatography
PEC: post-ERCP cholecystitis
PEP: post-ERCP pancreatitis
